# Visualizing energy landscapes with metric disconnectivity graphs

**DOI:** 10.1002/jcc.23643

**Published:** 2014-05-28

**Authors:** Lewis C Smeeton, Mark T Oakley, Roy L Johnston

**Affiliations:** 1 School of Chemistry, University of BirminghamEdgbaston, Birmingham, B15 2TT, United Kingdom

**Keywords:** collective variables, protein, coarse-grained models, software, Python

## Abstract

The visualization of multidimensional energy landscapes is important, providing insight into the kinetics and thermodynamics of a system, as well the range of structures a system can adopt. It is, however, highly nontrivial, with the number of dimensions required for a faithful reproduction of the landscape far higher than can be represented in two or three dimensions. Metric disconnectivity graphs provide a possible solution, incorporating the landscape connectivity information present in disconnectivity graphs with structural information in the form of a metric. In this study, we present a new software package, PyConnect, which is capable of producing both disconnectivity graphs and metric disconnectivity graphs in two or three dimensions. We present as a test case the analysis of the 69-bead BLN coarse-grained model protein and show that, by choosing appropriate order parameters, metric disconnectivity graphs can resolve correlations between structural features on the energy landscape with the landscapes energetic and kinetic properties.

## Introduction

The potential energy surface,*U*(**r**), of an *N* atom chemical system represents the potential energy as a function of 3*N* atomic coordinates. The topography of *U*(**r**), or energy landscape, determines its structure, kinetics, and thermodynamics^1^,^2^ and its analysis has proved useful in studying a range of physical systems and phenomena, including glasses,^3^ biomolecules,^4^–^6^ and clusters.^7^–^9^ For all but the simplest cases, *U*(**r**) has many more degrees of freedom than it is possible to visualize conventionally, making it impossible to assess the surface topography directly. One solution to the visualization problem is to partition the landscape into discrete regions, and then hierarchically cluster these regions according to some similarity measure. This clustering can then be represented as a tree-graph in either two or three dimensions (2D or 3D). There are a number of examples of hierarchical clustering methods in the literature, broadly based on either geometry, energetic barriers, or local ergodicity.

In geometrical clustering, regions are clustered according to their structural similarity, which is usually defined by the root-mean-square deviation (RMSd) between them. In this context, regions can either correspond to minima on *U*(**r**) 10 or points along a molecular dynamics trajectory.^11^,^12^ The structures are clustered either by an iterative process, by which each structure is joined to its nearest neighbor until only a single cluster remains,^11^ or clustering structures that are within a critical distance of one another.^10^

When clustering according to energetic barriers, the landscape is partitioned into basins of attraction whereby each point on the landscape *U*(**r**), is mapped onto a local minimum α, with coordinate, **r**_α_ by a steepest-descent path.^3^,^13^ Alternatively, the landscape can be partitioned using a lumping approach,^14^ in which energy thresholds are used to group connected regions below the threshold. The similarity measure used for hierarchical clustering is the barrier energy that separates any two regions. Starting from the energy of the global minimum, *U*_0_, regions are clustered together if they are separated by a barrier with an energy lying in the interval *U*_*i* + 1_ − *U*_*i*_, where *U*_*i* + 1_ − *U*_*i*_ + Δ*U* and Δ*U* is the width of the interval. This clustering is repeated until a particular energy threshold, *U*_*t*_, is reach, or all the minima are clustered together. Such graphs have come to be referred to as disconnectivity graphs,^13^,^15^ and have been used in a number of studies to visualize energy landscapes.^13^,^15^–^17^ Disconnectivity graphs retain both the energies of minima on *U*(**r**), and the barriers that separate them, making them a useful diagnostic in visually assessing the thermodynamic and kinetic behavior of a system.^18^,^19^ They can also be used to represent free-energy surfaces by estimating the vibrational entropy of minima and transition states on the landscape from the harmonic superposition approximation.^20^–^22^ Clustering landscapes by local ergodicity involves partitioning the landscape into basins about local minima. Equilibration between basins is determined by comparing forward and backward transition rates between states^23^ or the time-dependent probability distributions of connected basins.^24^

A weakness of the disconnectivity graph method is that it does not retain any structural information on the minima and thus neglects a large portion of the information contained in the energy landscape. Metric disconnectivity graphs capture some of this structure by defining a metric, and then calculating an order parameter from the metric for each minimum of interest on the landscape. The minima can then be plotted along a metric axis perpendicular to the energy axis. Metric information can be included in a number of other ways, such as by changing the color, or thickness of the nodes and edges.^25^,^26^ In this article, we will refer to metric disconnectivity graphs as those for which the nodes are organized along a metric axis. A judicious choice of metric captures overall structural trends in the system, while ignoring noisy or irrelevant information.

Here, we demonstrate the use of metric disconnectivity graphs, using several metrics, to visualize the energy landscapes of coarse-grained proteins. These disconnectivity graphs are plotted with our new energy landscape visualization package, PyConnect.^27^

## Methodology

### BLN model

Metric disconnectivity graph analysis was performed on a database of stationary points for a BLN model protein This database was generated with discrete path sampling^8^,^28^ as implemented in PATHSAMPLE.^29^ The BLN model^30^,^31^ is a coarse-grained, off-lattice protein model in which each protein residue is represented by one of three types of bead: hydropho**B**ic, hydrophi**L**ic, or **N**eutral. Here, we use a version of the BLN potential in which the interresidue distances and angles are restrained with stiff springs.^32^ The beads interacts with each other according to(1)
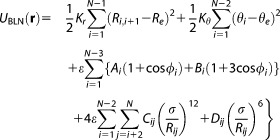
where *R*_*ij*_ is the distance between two beads *i* and *j*. The first term is a harmonic bond restraint with *K*_*r*_ = 231.2 ϵσ^−2^ and *R*_*e*_ = σ. The second term represents a harmonic angle constraint the *K*_*θ*_ = 20 rad^−2^ and θ_ϵ_ = 1.8326 rad. The third term takes into account torsional angles along the chain and is defined by four consecutive beads. If two or more beads are N, then *A* = 0 and *B* = 0.2, else *A* = *B* = 1.2. The fourth term represents long range, water-mediated hydrophobic interactions between nonbonded pairs. If both beads are B, then *C* = *D* = 1. If one residue is L and the other is L or B, then 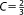 and *D* = −1. If either residue is *N*, then *C* = 1 and *D* = 0.^32^

Though other sequences exist and have been studied, we consider here BLN-69, which consists of 69 beads with the sequence^33^ B_9_N_3_(LB)_4_N_3_B_9_N_3_(LB)_4_N_3_B_9_N_3_(LB)_5_L. BLN-69 has been designed to exhibit a frustrated energy landscape, with a 6-strand β-barrel structure as its global minimum. The model has been shown to have a number of low-energy β-barrel-like structures, which differ from the global minimum by a chain slip along the length of the barrel,^4^ but are separated by large barriers. Such frustration is absent when considering the “Gō” version of the model (Gō-69), where attractive interactions between pairs of residues that are not in contact in the native state (i.e., the global minimum) are neglected.^34^,^35^

### Metric disconnectivity graphs

Disconnectivity graphs and metric disconnectivity graphs are plotted using PyConnect.^27^ The PyConnect package comprises two components: PCA, which calculates the principal components of molecular systems from PATHSAMPLE^29^ databases, and PyConnect, which constructs and displays metric disconnectivity graphs. Both of these programs were written in Python. The disconnectivity graphs are rendered with MatPlotLib,^36^ and users can choose to create disconnectivity graphs and metric disconnectivity graphs in 2D or 3D. PyConnect also provides some cosmetic features, including the ability to label minima, color minima according to an order parameter or according to their basin of residence. PyConnect can also be used to modify graphs interactively using the iPython^37^ virtual environment. In the disconnectivity graphs produced by PyConnect, the position of nodes and minima along the *x* axis are determined by algorithms similar to those used in DISCONNECT,^38^ another program for producing disconnectivity graphs from databases of minima and transition states. Full details of the algorithms used can be found on the PyConnect website.^27^ Two-dimensional metric disconnectivity graphs are plotted with the position of the minima on the *x* axis defined according to a metric. In 3D disconnectivity graphs, two metrics are used. The positions of nodes on the disconnectivity graphs are defined as the mean of the metrics for all minima connected to that node.

#### Native Contact Metric

The native contact metric evaluates for each minimum, *α*, the ratio *N*_α_/*N*_*NC*_, where *N*_*NC*_ is the number of native contact pairs, and *N*_α_ is the number of contact pairs in minimum *α* that are also present in the native conformation. Here, contacts are defined as those beads which are within 1.167σ of each other, excluding pairs that are within three beads of each other in the peptide sequence.^4^

Hydrogen bonding is important in protein folding, and native contact analysis can provide a useful analogy for coarse-grained protein models.*N*_α_/*N*_*NC*_ is commonly used as a progress variable in computational studies of protein folding to distinguish between the different degrees of partially folded protein.^39^

#### RMSd Metric

The RMSd, *d*_*α,β*_, measures the distance between the conformation of minimum α and *β*, **r**_*α*_ and **r**_*β*_ respectively, according to(2)
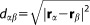


Invariance under global translations and rotations is implicit if structures are represented in internal coordinates. When working in Cartesian coordinates, the Kabsch algorithm^40^ was used to align structures to minimize *d*_*αβ*_. In the RMSd metric, *d*_*αβ*_ is calculated between the conformation of each minimum and the conformation of the global minimum, **r**_*GM*_.

#### Principal Component Metric

The principal component metric is based on principal component analysis (PCA), a statistical procedure used to analyze large, high-dimensional data sets, which is commonly used in dimensional reduction and, or when the relevant degrees of freedom in a data set are not clear.^41^ PCA attempts to reexpress a data set in terms of a new basis set, the principal components, which are a linear transform of the data sets original basis set. The principal components lie along the axes of greatest sample variance, with the first principal component, PC1, capturing the axis of greatest variance, the second principal component, PC2, capturing the axis of second greatest variance (orthogonal to the first) and so on.^42^

We performed PCA on the set of *N*_*sp*_ stable configurations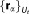, where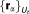 are all local minima connected to the global minimum below a certain threshold energy *U*_*t*_. The initial basis sets employed were the 3*N* dimensional external Cartesian basis set, {**e**_*i*_}, and an internal basis set of dihedral angles,{*ψ*_*i*_}. To remove the periodicity of {*ψ*_*i*_}, we used the sines and cosines of the internal dihedrals, {cos*ψ*_*i*_, sin*ψ*_*i*_}.^43^ Rotational and translational invariance of 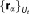 was enforced by implementing McLachlan's best fit procedure^44^;

1. A reference configuration defined as the ensemble average,〈**r**〉 of {**r**_*α*_} was calculated, where {**r**_*α*_} is the set of *N*_*sp*_ minima of interest, and where each configuration in {**r**_*α*_} has its centroid centered on the origin.
2. Define a new set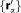, rotate each configuration about its origin to be as close to 〈**r**〉 as possible using the Kabsch algorithm, and thus minimize(3)
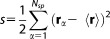
3. Replace {**r**_*α*_} with 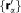.
4. Repeat steps 1–3 until the ensemble average converges to some threshold criterion.

In our study, we used the threshold criterion defined by Komatsuzaki et al.,^25^ *s* ≤ 10^−8^.

Hereafter, whether discussing Cartesian or internal coordinates, we define {**r**_*α*_} as the translation-free, rotation-free set of configurations, and {**q**_*i*_} as the basis set, where *i* is the coordinate index.

To perform PCA, we begin with defining the 3*N* × *N*_*sp*_ mean-centered configuration matrix, **R**(4)
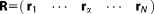
where each column of **R** is a 3*N* dimensional vector corresponding to a stable configuration in the set 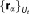. The PCs are the eigenvectors of the 3*N* × 3*N* covariance matrix, **C**(5)
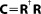
and are thus the basis set {**Q**_*i*_}, where *i* is the coordinate index, in which **C** is diagonalized. The PCs are calculated using the singular value decomposition method, which states that a 3*N* × *N*_*sp*_ configuration matrix, **R** can be written as the product(6)
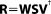
where **W** is the 3*N* × 3*N* matrix;(7)
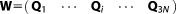
whose columns are the PCs of **C** and **S** is a 3*N* × *N*_*sp*_ matrix with diagonal elements, *S*_*ii*_, where (dropping the double index for clarity)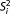 is the variance associated with **Q**_*i*_. The PCs are ordered so that **Q**_1_ has the greatest variance, **Q**_2_ has the second greatest variance, and so on. The *i*th principal component metric is calculated by transforming each member of 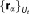 into the basis set of {**Q**_*i*_}, and using the value of the *i*th PC for each minimum as the order parameter.

One can visualize the PCs of a given 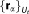, by choosing a reference structure, **r**_ref_, and adding the **Q**_*i*_ of interest to it(8)
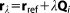
where *λ* is a progress variable.

#### Isomap Metric

The Isomap metric is based on the Isomap algorithm,^45^,^46^ a nonparametric, nonlinear dimensionality reduction technique. The aim of the Isomap algorithm is to define a low-dimensional embedding that as accurately as possible preserves geodesic distances between all pairs of points in the data cloud. The geodesic distance between a pair of points that lie on a manifold is the length of the shortest path between them that lies along that manifold. Isomap assumes that such a low-dimensional manifold exists, and that its shape can be estimated from the distribution of points in the data cloud. The Isomap algorithm approximates the geodesic distance between a given pair of points on the manifold by calculating the shortest possible path between them that can be found by stepping from one point to its neighbor.

We applied Isomap to the set of *N*_*sp*_ stable configurations 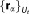 using the Isomap implementation in the scikit-learn machine learning package.^47^ As with the principal component metric, rotational and translational invariance of 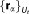 was enforced by implementing McLachlan's best fit procedure.

The Isomap algorithm works in three steps;

1. A weighted graph, *G*, of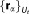 is built, where each conformation is a node and where the *k* nearest-neighbors of each conformation *α* are joined by an edge with weight *d*_*αβ*_. Isomap has been shown to be fairly robust to the choice of *k*,^46^ and in this study we took *k* = 15.
2. The shortest path between each conformation through the graph *G* is determined and a distance matrix, **D**, is computed, where *D*_*αβ*_ = min{*d*_*αβ*_, *d*_*αγ*_ + *d*_*γβ*_} for *γ* = 1, &, *N*_*sp*_. The elements *D*_*αβ*_, are the approximate geodesics between conformations *α* and *β*.
3. Classical multidimensional scaling is applied to the matrix *D*, producing a low-dimensional embedding of the conformational coordinates that best preserves geodesic distances on the manifold.

The *i*th Isomap metric corresponds to the *i*th embedded dimension of the low-dimensional manifold of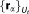.

## Results and Discussion

### BLN-69

For BLN-69, a database containing 141,835 minima and 173,692 transition states was used in the study. The first three Cartesian and dihedral principal components for the sets,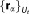, connected to the global minimum below energy *U*_*t*_, where −95.0ε ≥ *U*_*t*_ ≥ − 98.0ε are shown in Table 1.

**Table 1:** The variance captured by the first three principal components in Cartesian, 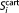, and dihedral,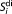, bases of the *N*_*sp*_ structures in the sublevel sets of minima below threshold energy *U*_*t*_ for BLN–69.

*U*_*t*_/ε	*N*_*sp*_	Cartesian PCA			Dihedral PCA		
		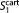	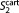	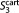	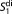	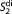	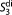
−95.0	6891	25.0	9.2	8.0	12.3	8.5	8.2
−95.5	5973	25.7	8.9	8.1	12.5	8.6	8.4
−96.0	5135	26.0	8.7	8.2	12.3	8.9	8.6
−96.5	4353	27.4	8.5	8.2	12.7	9.2	9.8
−97.0	1611	37.0	10.0	7.9	12.8	10.6	9.7
−97.5	561	21.2	19.0	10.8	15.1	13.7	11.9
−98.0	409	25.8	16.6	10.8	15.6	14.1	12.0

**Figure 1:**
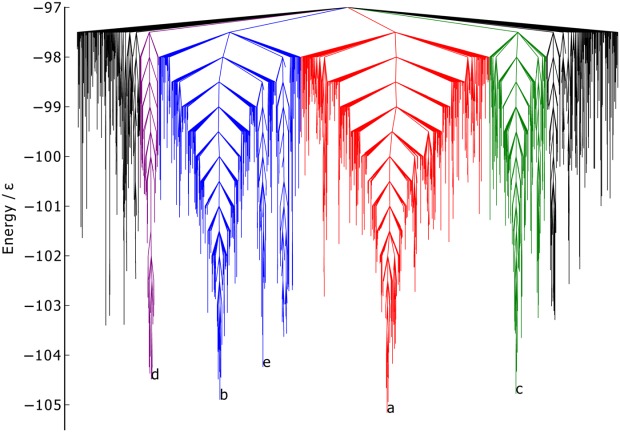
Disconnectivity graph of BLN-69,*U*_*t*_ = 97.0ε, *N*_*sp*_ = 1611. The color scheme is chosen to distinguish between energetic funnels. Labeled minima correspond to the global minimum and low-energy minima separated from the global minimum and one another by large kinetic barriers and are shown in Figure 2.

**Figure 2:**
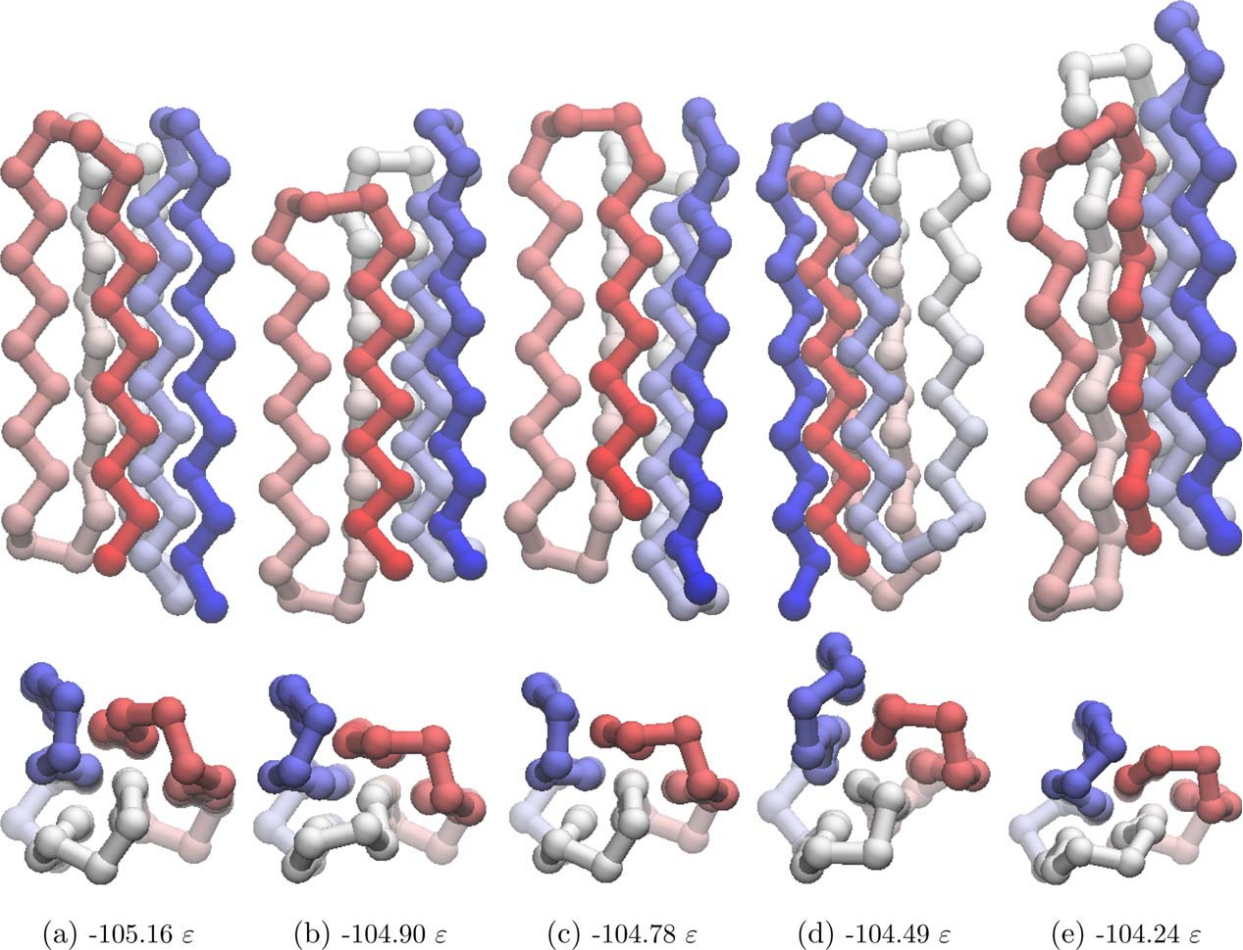
Structures of the minima labeled in Figure 1, corresponding to the global minimum, Figure 2a and low-energy minima separated from the global minimum and one another by large kinetic barriers, Figures 2b–2e. Energetic and structural details are provided in Table 2. The beads are colored from red to blue (N-terminus to C-terminus).

For the Cartesian PCs, PC1 captures significantly more of the variance than PC2 for all data sets considered. PC1 for the sublevel set of minima connected to the global minimum below *U*_*t*_ = −52.0ε has the largest fractional variance and therefore this threshold was selected for all disconnectivity graphs. The dihedral PCs have a more uniform variance distribution than the Cartesian PCs, with 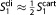, for all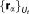 considered in both BLN-69 and Gō-69. The dihedral PCs are thus not appropriate metrics for studying these systems, and have not been used to create metric disconnectivity graphs. The set of minima where *U*_*t*_ = −97.0ε is represented as a disconnectivity graph in Figure 1. Figure 2 shows the low-energy minima labeled *a*–*e* in Figure 1. Minima *b*–*e* are all structurally similar to one another with each adopting compact β-barrel geometries and differing from global minimum *a* by either a chain-slip, chain-reptation, or twist in the turn regions, with further details given in Table 2.

**Table 2:** Energy above the global minimum, **ΔU**, fraction of native contacts, **N**_**α**_/**N**_**NC**_, RMSd from the global minimum and difference in PC1 and PC2 from the global minimum **ΔQ**_1_ and **ΔQ**_2_, respectively, for minima *b*–*e*.

Minimum	Δ*U*/ε	*N*_*α*_/*N*_*NC*_	RMSd/σ	ΔQ_1_/σ	ΔQ_2_/σ	Defect
*b*	0.26	0.85	0.41	−5.32	−1.25	Chain-slip
*c*	0.38	0.87	0.21	−0.02	−0.76	Reptation
*d*	0.67	0.86	0.40	−2.73	−3.51	Double chain-slip
*e*	0.92	0.77	0.54	−6.54	−2.61	Twist

**Figure 3:**
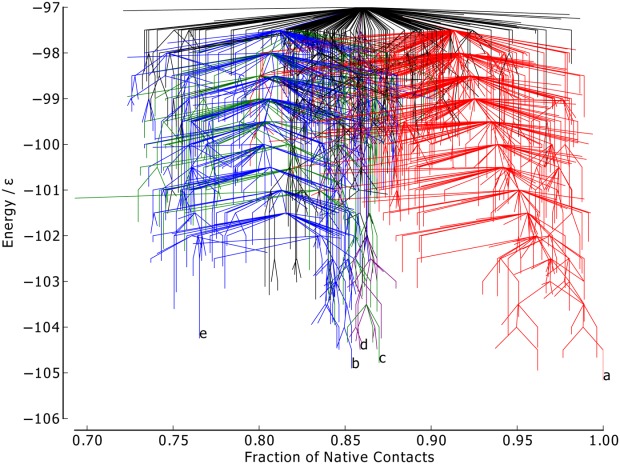
Metric disconnectivity graph of BLN-69, *U*_*t*_ = − 97.0ε, *N*_*sp*_ = 1611, with fraction of native contacts used as an order parameter. The color scheme and labels are as used in Figure 1. [Color figure can be viewed in the online issue, which is available at http://wileyonlinelibrary.com.]

The native contact metric (Fig. 3) splits the two largest funnels, with each having distinct fractions of native contacts (the mean fraction of native contacts for the funnels containing minima *a* and *b* is 0.91 and 0.81, respectively). Though the native contact metric differentiates between kinetically separated minima, it does not differentiate according to their energies. There are a number of unstable, high-energy minima with energetically unfavorable turns in the flexible *N* bead regions, but otherwise with almost all native contacts satisfied. Minimum *a* by definition satisfies all native contacts. Minima *b*–*d* are all very similar according to this metric, with each satisfying ≈ 85% of possible native contacts, in spite of their geometries being relatively dissimilar.

**Figure 4:**
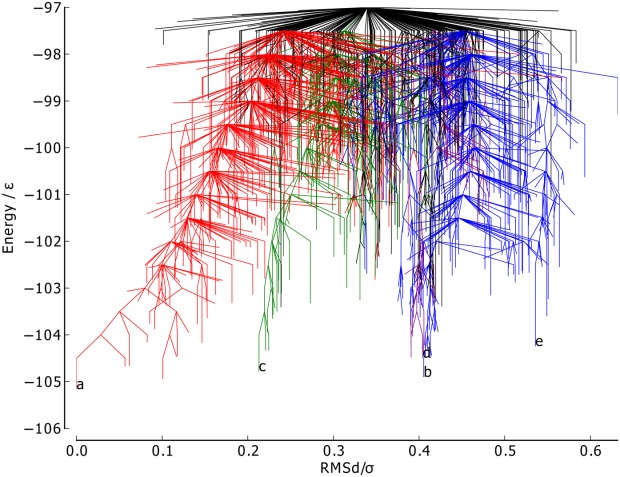
Metric disconnectivity graph of BLN-69, *U*_*t*_ = − 97.0ε, *N*_*sp*_ = 1611, with RMSd of each structure from the global minimum used as an order parameter in units of σ. The color scheme and labels are as used in Figure 1. [Color figure can be viewed in the online issue, which is available at http://wileyonlinelibrary.com.]

The RMSd metric (Fig. 4) is capable of distinguishing between the different major funnels on the surface, with each having its own mean value of the metric (mean RMSd for the funnels containing minima *a*, *c*, and *b* 0.91, and 0.81, respectively). There is also some relation to the minima energy in the green and red funnels, where lower energy corresponds to RMSd metric values closer to 0. The RMSd metric differentiates the basin minima into four groups, with minimum *c* being most similar to the global minimum, which is as expected from a visual inspection of the structures.

**Figure 5:**
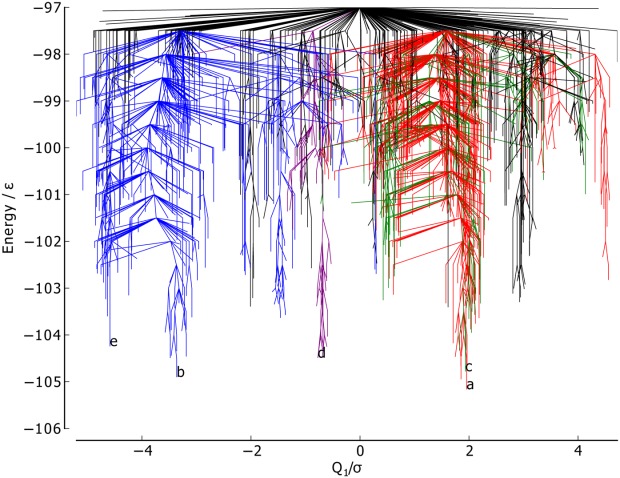
Metric disconnectivity graph of BLN-69,*U*_*t*_ = − 97.0ε, *N*_*sp*_ = 1611, with PC1 for 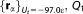, used as an order parameter in units of σ. The color scheme and labels are as used in Figure 1. [Color figure can be viewed in the online issue, which is available at http://wileyonlinelibrary.com.]

The PC1 metric (Fig. 5) splits the blue funnel (mean 3.29σ) from the red and green funnels, which sit on top of one another (mean 1.59σ and 1.60σ, respectively). The purple funnel lies at the boundary of the two, with a mean of −0.86σ. Minima *a* and *c* have almost identical values of *Q*_1_, while minima *d*, *b*, and *e* have increasingly dissimilar values. Given that PC1 corresponds to a chain-slip between the C and N termini, and that minima *d*, *b*, and *e* have chains that have shifted relative to the global minimum in the same direction, it gives confidence that PCA is capable of identifying structural features of the energy landscape.

**Figure 6:**
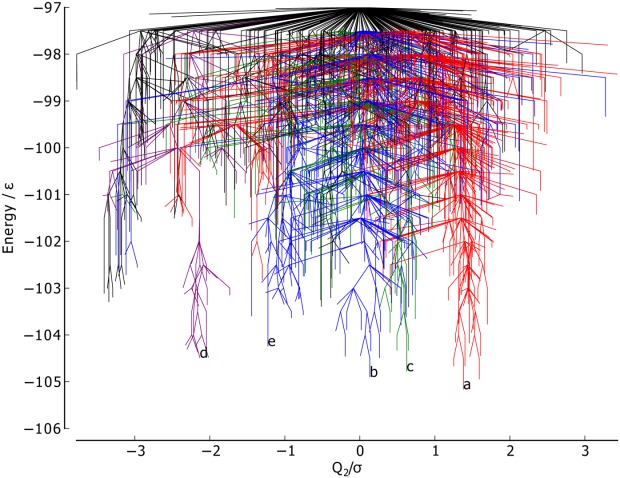
Metric disconnectivity graph of BLN-69, *U*_*t*_ = − 97.0ε, *N*_*sp*_ = 1611, with PC2 for 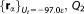, used as an order parameter in units of σ. The color scheme and labels are as used in Figure 1. [Color figure can be viewed in the online issue, which is available at http://wileyonlinelibrary.com.]

The PC2 metric (Fig. 6) does not reveal any obvious correlation between structure and energetics or kinetics, with no distinction made between the funnels and with the points reasonably evenly distributed along the order parameter. Thus, this PC corresponds to variations within all of the funnels rather than structural differences between the funnels.

**Figure 7:**
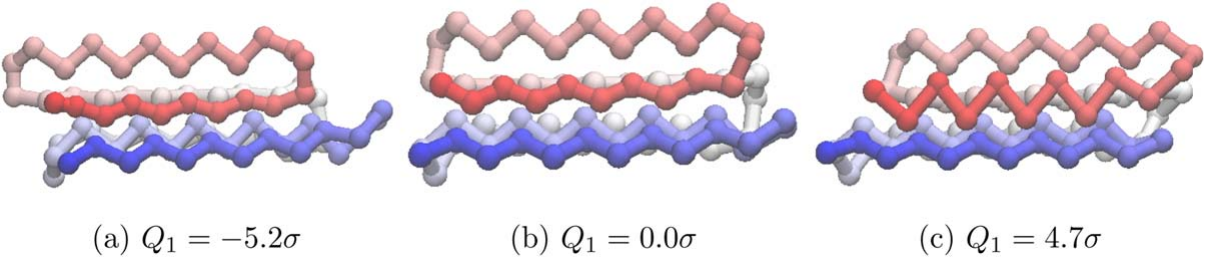
Different values of *Q*_1_ for *U*_*t*_ = −97.0ε projected onto the structure of the global minimum of BLN-69. For the global minimum, *Q*_1_ = 1.96σ. The beads are colored from red to blue (N-terminus to C-terminus). An animated version of this projection is available as Supporting Information. [Color figure can be viewed in the online issue, which is available at http://wileyonlinelibrary.com.]

**Figure 8:**
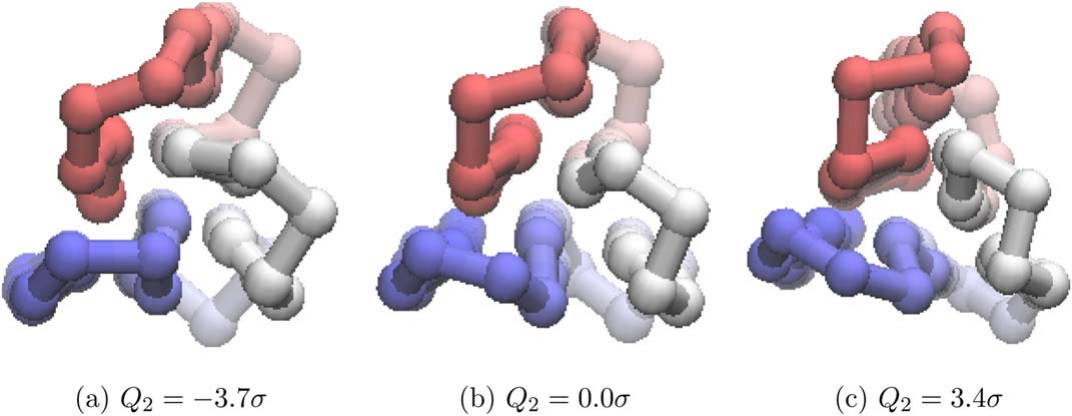
Different values of *Q*_2_ for *U*_*t*_ = −97.0ε projected onto the structure of the global minimum of BLN-69. For the global minimum, *Q*_2_ = −5.5σ. The beads are colored from red to blue (N-terminus to C-terminus). An animated version of this projection is available as Supporting Information. [Color figure can be viewed in the online issue, which is available at http://wileyonlinelibrary.com.]

The progression of PC1 of 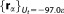 from *λ* = −5.2σ to *λ* = 4.7σ (Fig. 7) corresponds to a chain-slip between the C and N termini. The progression of PC2 of 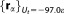 from *λ* = −3.7σ to *λ* = −3.4σ (Fig. 8) corresponds to a twisting of the internal chain sequences.

**Figure 9:**
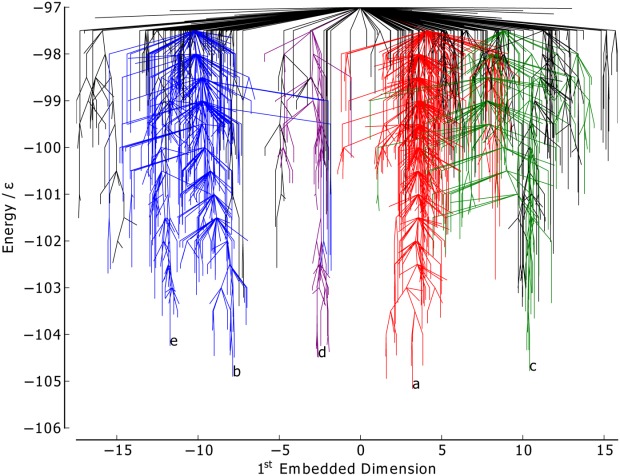
Metric disconnectivity graph of BLN-69, *U*_*t*_ = −97.0ε, *N*_*sp*_ = 1611, with the first embedded dimension for 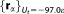 from Isomap analysis used as an order parameter in units of σ. The color scheme and labels are as used in Figure 1. [Color figure can be viewed in the online issue, which is available at http://wileyonlinelibrary.com.]

The first embedded dimension of the Isomap metric (Fig. 9) clearly differentiates between all the colored funnels on the landscape. The mean value of the blue, purple, red, and green funnels are −10.23σ, −2.96σ, 4.12σ, and 8.80σ, respectively. The structure of the graph is similar to the PC1 graph (Fig. 5), with the order of the colored funnels and labeled low-energy minima along the metric axis matching. The agreement between these two metrics suggests that the first embedded dimension of the Isomap metric is fairly linear, and that, as with the PC1 metric, it corresponds to a chain-slip between the C and N termini.

**Figure 10:**
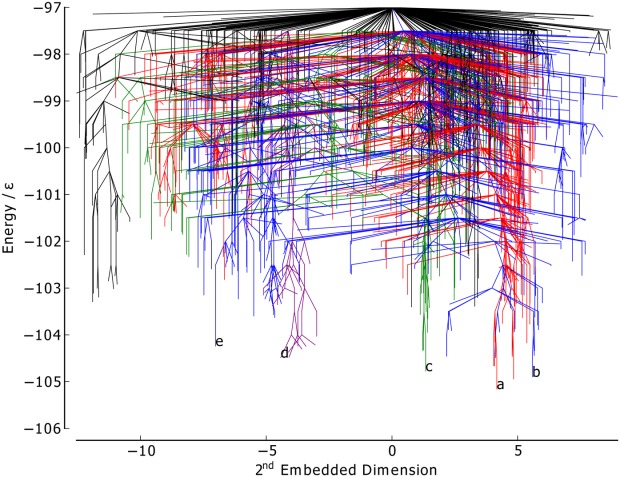
Metric disconnectivity graph of BLN-69,*U*_*t*_ = −97.0ε, *N*_*sp*_ = 1611, with the second embedded dimension for 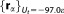 from Isomap analysis used as an order parameter in units of σ. The color scheme and labels are as used in Figure 1. [Color figure can be viewed in the online issue, which is available at http://wileyonlinelibrary.com.]

As with the PC2 metric, the disconnectivity graph for the seconded embedded dimesnion of the Isomap metric (Fig. 10) is difficult to interpret. The overlapping of the colored funnels suggests that the second embedded dimension corresponds to some structural variation common to each funnel.

The information in Figures 5 and 6 is visualized on a single 3D metric disconnectivity graph of 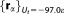 projected onto the plane of maximal variance in Figure 1. The plot shows 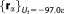 for BLN-69 plotted against its first two principal components. Clear separation of minima *a*–*e* is discernible in this 3D metric disconnectivity graph.

**Figure 11:**
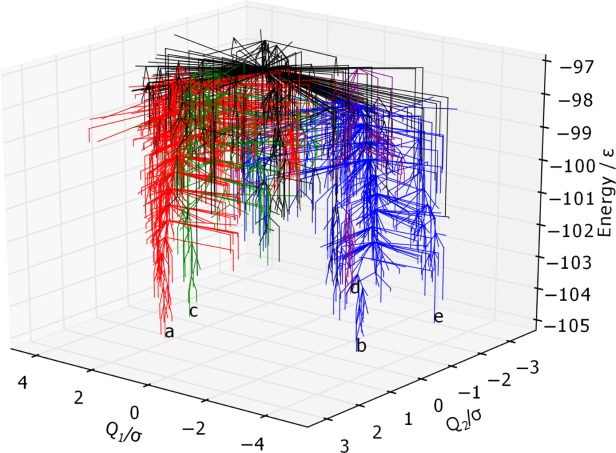
3D metric disconnectivity graph of BLN-69,*U*_*t*_ = −97.0ε, *N*_*sp*_ = 1611, plotted with the first two principal components of 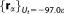, *Q*_1_ and *Q*_2_, used as order parameters in units of σ. The color scheme and labels are as used in Figure 1. [Color figure can be viewed in the online issue, which is available at http://wileyonlinelibrary.com.]

### Gō-69

For Gō-69, a database containing 75,666 minima and 113,101 transition states was used. The first three Cartesian principal components for the sets, 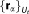, connected to the global minimum below energy *U*_*t*_, where −52.0ε ≥ *U*_*t*_ ≥ − 58.0ε are shown in Table 3.

**Table 3:** The variance captured by the first three principal components in Cartesian, 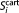, and dihedral,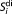, bases of the *N*_*sp*_ structures in the sublevel sets of minima below threshold energy *U*_*t*_ for Gō-69.

*U*_*t*_/ε	*N*_*sp*_	Cartesian PCA			Dihedral PCA		
		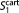	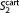	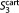	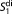	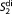	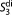
−52.0	5529	38.6	14.3	12.6	14.4	10.6	7.3
−53.0	4364	38.0	13.7	12.0	13.7	11.1	7.7
−54.0	3188	24.5	16.0	8.0	13.3	11.6	8.2
−55.0	2386	24.4	16.0	8.1	13.3	11.3	8.7
−56.0	1691	23.7	15.7	7.6	14.0	12.1	9.4
−57.0	1185	21.6	16.3	7.7	13.9	12.7	10.4
−58.0	739	21.5	15.3	7.7	14.9	12.9	11.4

As with the Cartesian PCs of BLN-69, PC1 captures significantly more of the variance than PC2 for all data sets considered. PC1 for the sublevel set of minima connected to the global minimum below *U*_*t*_ and *U*_*t*_ = − 53.0ε, have the largest fractional variance, though these large variances are due to a comparatively small number of unstable, high-energy minima in which one end of the chain has peeled away from the barrel and become unbound. For these systems, PC1 is no longer representative of the distribution of minima on *U*_**r**_. For this reason, we consider the sublevel set of minima connected to the global minimum below *U*_*t*_ = − 52.0ε, for which all the minima have densely packed geometries. This set is represented as a disconnectivity graph in Figure 2. The results for the Isomap metric were fairly ambiguous for Gō, with no obvious pattern correlation between the embedded dimensions and the kinetic or energetic structure of the graph, so they have not been included in this work.

**Figure 12:**
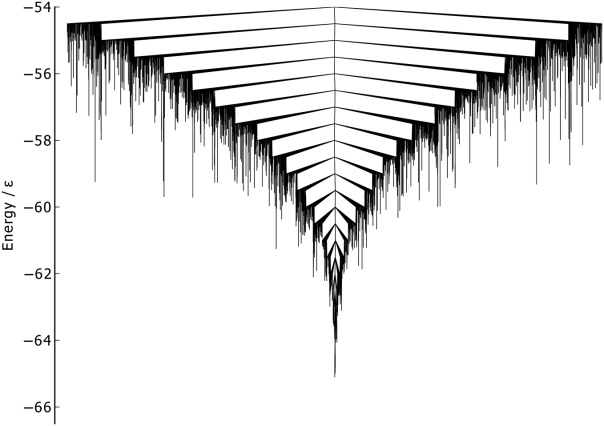
Disconnectivity graph of Gō-69, *U*_*t*_ = − 54.0ε, *N*_*sp*_ = 3189.

**Figure 13:**
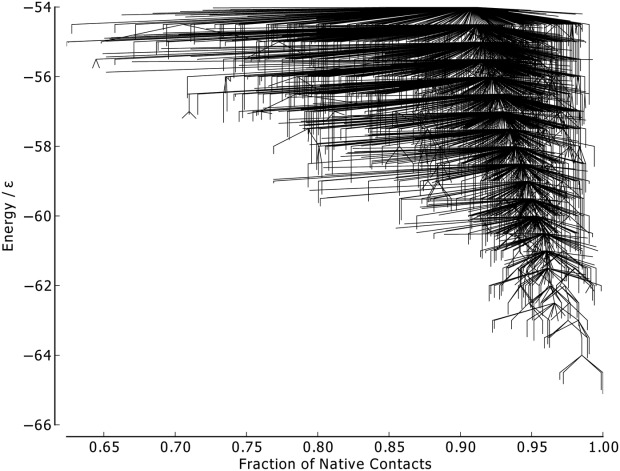
Metric disconnectivity graph of Gō-69, *U*_*t*_ = − 54.0ε, *N*_*sp*_ = 3189, with fraction of native contacts used as an order parameter.

As there is only a single funnel on the Gō-69 landscape, there are no large kinetic barriers for any of the metrics to differentiate between. The native contact metric (Fig. 13) is able to partially distinguish between the structures of high- and low-energy minima. As with BLN-69, minima across the whole energy range examined were able to satisfy nearly full native contacts, including unstable, high-energy minima with energetically unfavorable turns in the flexible *N* bead regions. The converse is not true; however, as all low-energy minima have a high number of native contacts and low numbers of native contacts are only found for high-energy minima.

**Figure 14:**
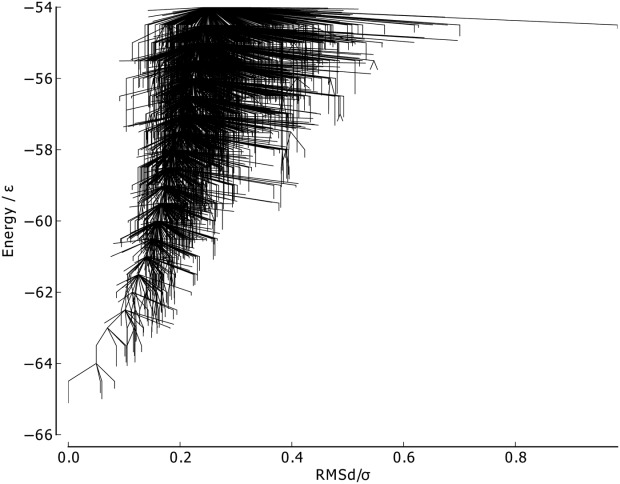
Metric disconnectivity graph of Gō-69, *U*_*t*_ = − 54.0ε, *N*_*sp*_ = 3189, with RMSd of each structure from the global minimum used as an order parameter in units of σ.

For the RMSd metric (Fig. 14), similar behavior to BLN–69 is exhibited, albeit with a single funnel, with RMSd from the global minimum increasing with increasing energy.

**Figure 15:**
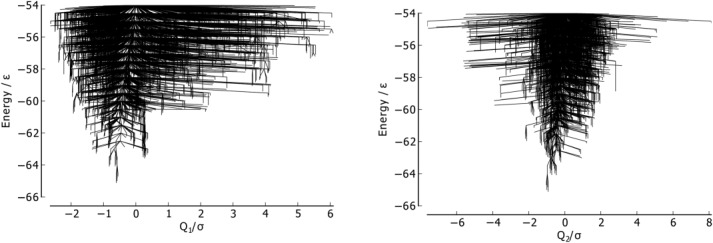
Metric disconnectivity graphs of Gō-69, *U*_*t*_ = − 54.0ε, *N*_*sp*_ = 3189, with PC1 (left) and PC2 (right) for 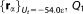 and *Q*_2_, used as order parameters in units of σ.

**Figure 16:**
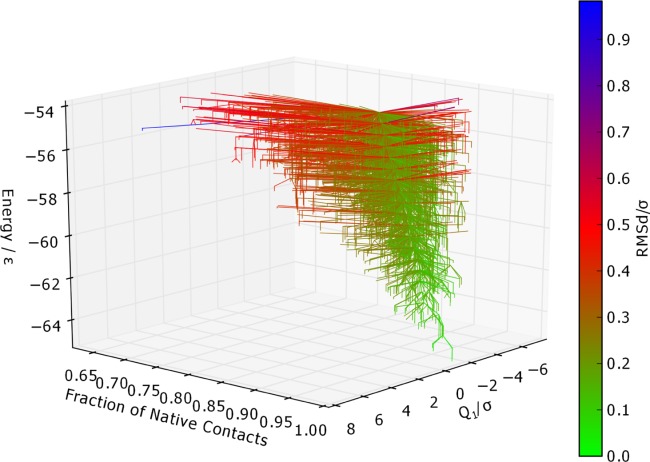
Metric disconnectivity graph of Gō-69, *U*_*t*_ = − 54.0ε, *N*_*sp*_ = 3189, with PC1 for 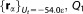, and fraction of native contacts used as order parameters, and colored according to RMSd of each structure from the global minimum. *Q*_1_ and RMSd are in units of σ. [Color figure can be viewed in the online issue, which is available at http://wileyonlinelibrary.com.]

The metric disconnectivity graphs in Figure 15 use PC1 and PC2 as order parameters. In the PC1 graph, the majority of minima are centered about the global minimum, with a smaller number of high-energy minima extending to higher values of PC1. PC1 and the fraction of native contacts are well-correlated, as can be seen in the 3D metric disconnectivity graph (Fig. 16). The PC2 metric orders all but a few minima tightly in a rough column about *Q*_2_ ≈ 0.1. Those unstable, higher energy minima that are not in that column are structures with a partly unbound C-terminus chain-portion.

The use of color allows an additional metric to be included on a metric disconnectivity graph. For example, Figure 6 shows the PC1, native contact, and RMSd metrics for Gō-69.

Figure 7 shows the progression of PC1 of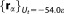 from *λ* = − 2.6σ to *λ* = 6.1σ, with a view along the axis of the barrel and corresponds to a sweeping action of the red chain-portion across the face of the white chain-portion.

**Figure 17:**
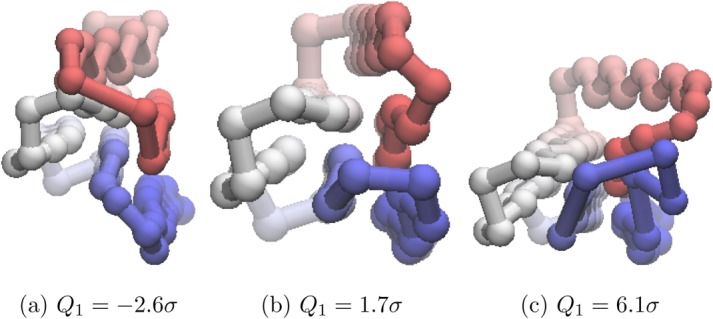
Different values of *Q*_1_ for *U*_*t*_ = − 54.0ε projected onto the structure of the global minimum of Gō-69. For the global minimum, *Q*_1_ = −0.6σ. The beads are colored from red to blue (N-terminus to C-terminus). An animated version of this projection is available as Supporting Information. [Color figure can be viewed in the online issue, which is available at http://wileyonlinelibrary.com.]

**Figure 18:**
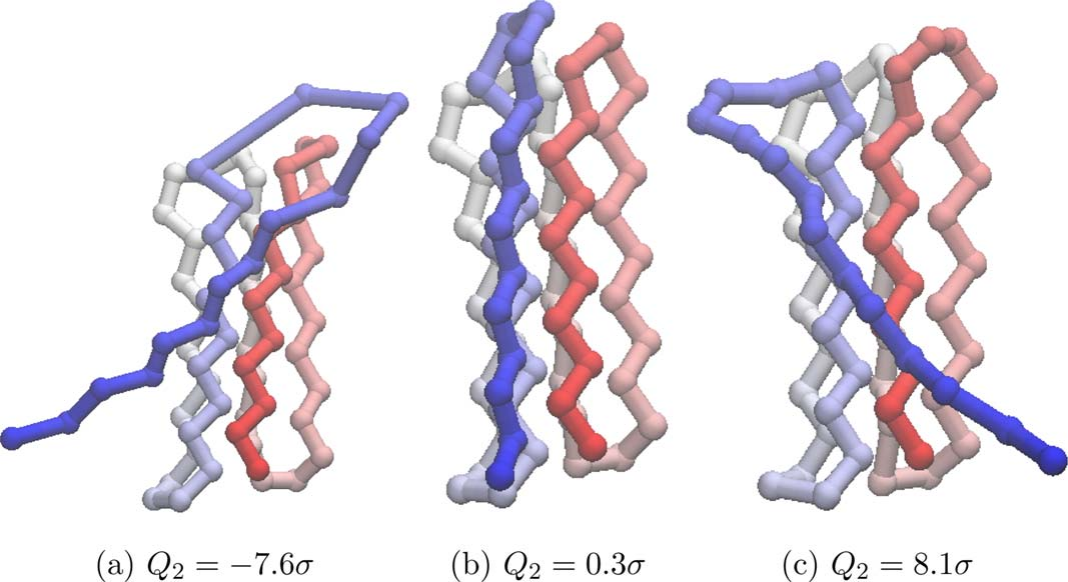
Different values of *Q*_2_ for *U*_*t*_ = − 54.0ε projected onto the structure of the global minimum of Gō-69. For the global minimum, *Q*_2_ = −0.9σ. The beads are colored from red to blue (N-terminus to C-terminus). An animated version of this projection is available as Supporting Information. [Color figure can be viewed in the online issue, which is available at http://wileyonlinelibrary.com.]

Figure 18 shows the progression of PC2 of 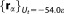 from *λ* = −7.6σ to *λ* = 8.1σ, and corresponds to a “can-can” like sweeping motion of the free C-terminus end of the chain.

## Conclusions

In this study, we have demonstrated how an appropriate order parameter can elucidate the connection between structures in the energy landscape of BLN-69 and Gō-69, such as funnels, with certain structural motifs of the protein, including chain slips and twists in the turn regions. However, there are still shortcomings to the metrics proposed. Fraction of native contacts and RMSd metrics relied on having prior knowledge of the system. PCA provides a means to study systems without resorting to chemical intuition, but still assumes that the point cloud is approximately linear, and cannot be directly implemented for angular coordinates. Also, it considers all structures to be of equal importance, regardless of energy, leading to situations such as with Gō-69, where all the variance in structure was provided by a small number of high energy, unstable minima. Isomap allows one to discern low-dimensional, nonlinear manifolds in the data, and does not make the same assumptions of linearity as PCA. This is clearly a successful strategy, with Isomap distinguishing between the different kinetic structures on the landscape. A useful feature of PCA is the ease with which one can project the principal components back into the original space, making it possible to visualize what these directions correspond to. In principal, it should be possible to do the same with Isomap, projecting the approximate geodesics of the manifold back into the original space, though we have not implemented this in the work presented here. Other nonlinear dimensionality reduction methods exist in the literature, such as sketch-map,^48^ locally scaled diffusion map,^46^,^49^ and spectral methods,^50^ which are good candidate metrics for further study.

Equally, though the data produced by PyConnect is of a high-quality, the data analysis is still fairly qualitative, and further efforts are being taken to quantify the observations, such as using graph-theoretic techniques to analyze and compare tree graphs.^51^

Future work should also focus on investigating more realistic, small protein systems, such G-protein^52^ or cyclic peptides.^5^,^22^
